# Evaluation of hepatitis C viral RNA persistence in HIV-infected patients with long-term sustained virological response by droplet digital PCR

**DOI:** 10.1038/s41598-019-48966-9

**Published:** 2019-08-29

**Authors:** Mario Frías, Antonio Rivero-Juárez, Francisco Téllez, Rosario Palacios, Álvaro Jiménez-Arranz, Juan A. Pineda, Dolores Merino, María Amparo Gómez-Vidal, Inés Pérez-Camacho, Ángela Camacho, Antonio Rivero

**Affiliations:** 1 Unidad de Enfermedades Infecciosas. Instituto Maimónides de Investigación Biomédica de Córdoba (IMIBIC), Hospital Universitario Reina Sofía de Córdoba, Universidad de Córdoba, Córdoba, Spain; 2 Unidad Gestión Clínica Enfermedades Infecciosas, Hospital La Línea, AGS Campo de Gibraltar, Cádiz, Spain; 3 Unidad de Enfermedades Infecciosas. Hospital Universitario Virgen de la Victoria, Complejo Hospitalario Provincial de Málaga, Málaga, Spain; 4 Unidad de Genómica. Instituto Maimónides de Investigación Biomédica de Córdoba (IMIBIC), Hospital Universitario Reina Sofía, Universidad de Córdoba, Córdoba, Spain; 5 Unidad de Enfermedades Infecciosas y Microbiología, Hospital Universitario de Valme, Instituto de Biomedicina de Sevilla (iBiS), Sevilla, Spain; 6 Unidad de Enfermedades Infecciosas, Hospitales Juan Ramón Jiménez e Infanta Elena de Huelva, Huelva, Spain; 7 Unidad de Enfermedades Infecciosas, Complejo Hospitalario de Jaén, Jaén, Spain; 8 Unidad de Enfermedades Infecciosas, Hospital de Poniente, El Ejido, Spain

**Keywords:** Molecular biology, Medical research

## Abstract

Several studies have reported the persistence of HCV RNA in liver and/or peripheral blood mononuclear cells (PBMCs) in spite of undetectable viremia in patients who have achieved sustained virological response (SVR). This event, defined as occult HCV infection, remains controversial and low titers of persistent virus may be underestimated because it has not yet been analyzed by a highly sensitive test such as droplet digital PCR (ddPCR). This method provides an alternate ultra-sensitive detection technique for very low numbers of copies of viral RNA or DNA. The aim of this study was to evaluate the persistence of HCV in HIV-coinfected patients with long-term SVR using ddPCR. For each patient, the presence of HCV RNA in serum and PBMCs at baseline was determined by nested RT-ddPCR. Patients with HCV RNA in PBMCs at baseline were followed until the end of the study. One hundred and twenty-three patients were analyzed for persistence of HCV RNA in serum and PBMCs. Persistence of HCV was not found in serum in any patient. HCV RNA was detected in PBMCs in one patient (0.81%; 95% CI: 0.04–3.94) and resolved spontaneously during follow-up. Persistence of HCV RNA in PBMCs is not a common event in HIV/HCV co-infected patients with long-term SVR evaluated by RT-ddPCR.

## Introduction

The objective of chronic HCV infection (CHC) therapy is to achieve sustained virological response (SVR), defined as absence of HCV RNA in serum at twelve or twenty-four weeks after successful treatment^1^. In the context of HCV infection, therefore, SVR is synonymous with cure, and an HCV RNA determination after SVR is only recommended if the patient has ongoing risk of HCV reinfection^1^. Several studies however have reported that the persistence of HCV RNA in the liver and/or peripheral blood mononuclear cells (PBMCs) in patients who have achieved SVR can lead to late relapses^2–6^. This unusual event is recognized as occult HCV infection and defined as the presence of HCV RNA in liver tissue or PBMCs despite undetectable viremia^7^. This situation remains controversial as several studies have been unable to confirm the presence of HCV RNA in PBMCs or liver tissue in patients with long-term viral clearance^8–10^. Low titers of persistent virus may be underestimated as this event has not been confirmed with highly sensitive tests with lower limits of detection than reverse transcriptase polymerase chain reaction (RT-PCR) or transcription-mediated amplification (TMA).

Droplet digital PCR (ddPCR) represents an alternative technique, providing ultra-sensitive detection of very low numbers of copies of viral RNA or DNA^11–14^. In the context of viral infection, ddPCR has been demonstrated to be useful in the measurement of HIV reservoirs^15,16^. Given that viral persistence in SVR patients remains controversial, the use of ddPCR may be significant for detecting residual HCV RNA in this population. The objective of our study therefore was to use ddPCR to evaluate the persistence of HCV in HIV-coinfected patients with long-term SVR.

## Material and Methods

### Study populations

HIV-infected patients in follow-up at seven reference hospitals in Andalusia (southern Spain) between January 2015 and January 2018 were included in this prospective longitudinal study. Patients included had to fulfill the following inclusion criteria: (i) treatment-induced SVR, defined as the absence of detectable serum HCV RNA 24 weeks after end of treatment; (ii) at least 12 months of SVR before study inclusion; (iii) annual undetectable serum HCV RNA levels by qRT-PCR (detection limit set at 15–20 IU/mL) after achieving SVR until inclusion in the study; (iv) annual liver stiffness measurements after achieving SVR. Patients with ongoing risk of HCV reinfection were excluded.

### Follow-up and variable collection

Using density gradient centrifugation, PBMCs were isolated from whole blood and serum samples collected from all patients (Ficoll-Hypaque). To stabilize RNA, isolated PBMCs and serum samples were treated with RNAlater Solution (Thermo Fisher Scientific, USA) in a 1:1 concentration and cryopreserved at −80 °C.

Nested RT-ddPCR was used to determine the baseline presence of HCV RNA in serum and PBMCs in all patients. Those patients with baseline presence of HCV RNA in PBMCs were followed until the end of the study. Follow-up consisted of an annual determinations of HCV RNA in both PBMCs and serum.

The liver stiffness (LS) records of all patients were collected from achievement of SVR until the end of the study. The cutoff values of LS used to determine liver fibrosis stage in this study were as follows: <6.5 kPa (F0-F1, minimal or absent fibrosis); 6.5–9.4 kPa (F2, significant fibrosis); 9.5–14.5 kPa (F3, advanced fibrosis) and ≥14.6 kPa (F4, cirrhosis)^17^. Patients whose liver fibrosis increased by at least one stage after achieving SVR were considered to be liver fibrosis progressed. Patients with no identifiable cause of liver fibrosis progression other than HCV infection underwent liver biopsy, and PCR was performed to detect HCV RNA in liver tissue. Patients with HCV RNA present in liver tissue were followed up.

The primary outcome of the study was occult HCV infection, defined as the presence of HCV RNA in PBMCs and/or liver tissue, in patients with an undetectable HCV viral load in serum by ddRT-PCR. From the achievement of SVR until the end of follow-up, possible variables related to persistence of HCV RNA in HIV patients were collected, including: (i) HCV infection-related variables: type of HCV therapy, duration of therapy, HCV genotype, baseline HCV viral load, and liver stiffness; (ii) HIV infection-related variables: CD4^+^ count, HIV RNA, AIDS criteria and use of combination antiretroviral therapy (cART); (iii) demographic and clinical variables: gender, age, alanine aminotransferase (ALT) and aspartate aminotransferase (AST). (iv) risk practices for HCV reinfection.

### Nested RT-ddPCR protocol

Viral RNA was extracted from the PBMC samples using the commercial RNeasy plus universal kit (Qiagen, Hilden, Germany). Viral RNA was extracted from serum with the QIAamp minElute virus spin kit (Qiagen, Hilden, Germany). Both extractions were performed using automated QIAcube procedures (QIAgen, Hilden, Germany). Two rounds of PCR were performed for HCV RNA detection, both using primers described previously: sense 5′-CTTCACGCRGAAAGCGYCTA3′ and antisense 5′-CAAGCACCCTATCAGGCAGT-3′ as outer primers, and sense 5′-GCGTTAGTAYGAGTGTYG-3′ and antisense 5′-CRATTCCGGTGTACTCAC-3 as inner primers^18^. These primers amplify the core region of the HCV genome for four viral genotypes (1, 2, 3 and 4).

The first round of PCR was performed with the iTaq Universal Probes One-Step Kit (Bio-rad, USA). The 20 µL reaction mix contained 10 µL of iTaq universal probes reaction mix (2×), 900 nM of each primer, 0.5 µL of iScript reverse transcriptase and 9.14 µL of template RNA. A Biorad C100 thermal cycler (Biorad; CA, USA) was used, and the cycling conditions were: 50 °C for 10 min, 95 °C for 3 min, and 45 cycles at 95 °C for 20 s, 55 °C for 30 s, and 60 °C for 20 s. The amplified product of 246-bp was then purified with the QIAquick PCR Purification Kit (QIAgen, Hilden, Germany). In the second PCR round, each reaction mix contained 10 µL of ddPCR™ Supermix for Probes (Bio-Rad, USA), 900 nM of each primer, 250 nM of FAM-labeled HCV probe (5′-FAM-CCGCAGACCACTATGGCTC-BHQ1-3′), and 9.35 µL of amplified product from the first round of PCR in a final volume of 20 μL. The length of the second-round PCR amplified product was 89-bp. Nuclease-free water (Sigma-Aldrich Inc., USA) was used as negative control, and both were processed in parallel with cDNA synthesis and analyzed directly by ddPCR. Serum and PBMC samples of patients chronically infected with HCV genotype 1 (serum viral load: 11,909,700 IU/mL), genotype 3 (serum viral load: 4,577,570 IU/mL), genotype 4 (serum viral load: 8,450,166 IU/mL) were used as positive controls, in a 1/200 dilution.

The ddPCR reaction mixes were placed in the Bio-Rad QX200™ Droplet Generator and droplets generated in accordance with the manufacturer’s instructions. The droplets were transferred to a 96-well PCR plate and sealed with Bio-Rad PX1™ PCR Plate Sealer, following the manufacturer’s instructions. Amplification was performed on a Bio-Rad C1000 Touch™ Thermal Cycler at the following cycling temperatures: 95 °C for 10 min, followed by 40 cycles at 94 °C for 30 s and at 55.5 °C for 1 minute and 1 cycle at 98 °C for 10 min, ending with a hold step at 4 °C. After PCR, the plate was immediately read on the Bio-Rad QX200™ Droplet Reader, where the droplets were analyzed.

Use of ddPCR gives an absolute count of genome copies of HCV in PBMCs without the use of standard curves. Fluorescence intensity in every well was analyzed with Bio-Rad QuantaSoft analysis software v. 1.7. After defining the threshold based on fluorescence amplitudes, the droplets were classified as positive or negative. The number of positive and negative droplets was used to calculate the target concentration. This concentration was estimated assuming a Poisson distribution. The QuantaSoft analysis software determined the target concentration by adjusting it to a Poisson algorithm. The formula used by the QuantaSoft software was: [C = -ln(Nneg/N)/Vdroplet], where: C = copies per droplet; Nneg = number of negative droplets; N = total number of droplets; Vdroplet = volume of droplet (0.85nL according to the manufacturer’s instructions).

### HCV antigenomic strand determination

PBMC samples positive by ddPCR were also analyzed for a negative-strand HCV determination. PCR was performed using the iTaq Universal Probes One-Step Kit (Bio-rad, USA). The sense and antisense primers used were 5′-AGACTCACTCCCCTGTGAGGAA-3′ and 5′- TGAGTGCACGGTCTACGAGACCTC-3′ respectively. The 20 µL reaction mix contained 10 µL of iTaq universal probes reaction mix (2×), 500 nM of each primer, 0.5 µL of iScript reverse transcriptase and 8.5 µL of template RNA. A thermal cycler Biorad C100 (Biorad; CA, USA) was used and the following thermal cycling conditions applied: 50 °C for 10 min, 95 °C for 3 min and 45 cycles at 95 °C for 20 s, 67 °C for 30 s, and 60 °C for 20 s. The amplified 311 bp product was then purified by QIAquick PCR Purification Kit (QIAgen, Hilden, Germany).

### Statistical analysis

Continuous variables were expressed as means (standard deviation) or median and quartiles (Q1-Q3). Categorical variables were expressed as numbers of cases and percentages. Analyses were carried out using the statistical software package SPSS version 18.0 (IBM Corporation, Somers, NY, USA) and GraphPad Prism version 6 (Mac OS X version; GraphPad Software, San Diego, CA, USA).

### Ethical statement

This study was designed and performed according to the Helsinki Declaration. All patients signed an informed consent form. The CEIC (Clinical Trial and Ethical Committee) of the Hospital Universitario Reina Sofía de Córdoba, the coordinating Hospital, approved the study protocol.

## Results

### Study population

One hundred and twenty-three HIV-infected patients with treatment-induced SVR were included in the study. The median time from SVR was 51 months (IQR: 27–76). The distribution of patients by time from SVR was: 12–24 months, n = 27 (21.9%); 25–36 months, n = 15 (12.2%); 37–48 months, n = 14 (11.4%), 49–60 months, n = 14 (11.4%) and more than 60 months, n = 53 (43.1%). The main patient characteristics are summarized in Table 1.

**Table 1:** Main baseline patient characteristics.

Clinical characteristics	Condition	N = 123
Age (years), median (IQR)	—	51 (48–55)
Sex, n (%)	Male	102 (82.9)
	Female	21 (17.1)
HIV viral load, n (%)	Undetectable	119 (96.7)
	Detectable	4 (3.3)
CD4 count at inclusion study (cell/mL), median (IQR)	—	560 (396–726.5)
Use of ART, n (%)	Yes	122 (99.2)
	No	1 (0.8)
HCV genotype, n (%)	1	59 (48)
	2	4 (3.2)
	3	54 (43.9)
	4	6 (4.9)
Type of HCV therapy^a^	Peg-IFN + RBV	102 (82.9)
	DAA^b^ + Peg-IFN + RBV	20 (16.3)
	IFN-free	1 (0.8)
Liver stiffness (kPa) at inclusion in study, median (IQR)	—	6 (4.7–7.7)
Liver stiffness (kPa) at SVR, median (IQR)	—	7.2 (5.4–11.7)
Time from SVR (months), median (IQR)	—	51 (27–76)

Interquertile range (IQR); n (number of cases); human immunodeficiency virus (HIV); antiretroviral therapy (cART); hepatitis C virus (HCV); pegylated interferon (Peg-IFN); ribavirin (RBV); direct-acting antiviral (DAA).

^a^HCV therapy which induced SVR;

^b^daclatasvir (n = 7); telaprevir or boceprevir (n = 13).

### Evaluation of low-titer persistent HCV in serum and PBMCs

**Figure 1:**
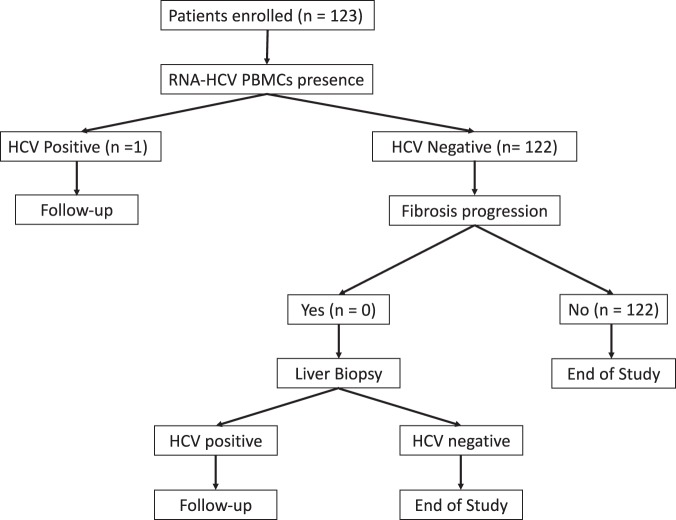
Flow chart of patients evaluated in the study.

The flow chart of patients in the study is shown in Fig. 1. One hundred and twenty-three patients were evaluated for HCV persistence in serum and HCV RNA was detected in none. The persistence of HCV RNA was then analyzed in PBMCs and was detected in 1 patient (0.81%; 95%CI: 0.04–3.94).

### Liver fibrosis progression

The median LS of included patients at the time of SVR was 7.2 kPa (IQR: 5.4–11.7). The median LS value at inclusion was 6 kPa (IQR: 4.7–7.7). None of the patients had criteria for fibrosis progression (increased liver stiffness) between achieving SVR and inclusion in the study and none therefore fulfilled the criterion for liver biopsy. Of the patients included, 14.6% had a liver stiffness score of over 14.5 kPa at achievement of SVR and the percentage of those with liver stiffness values of 14.5 kPa or more decreased to 6.5% at the start of the study.

### Follow-up of patient with occult infection

Of all the patients included in the study, only 1 patient fulfilled the criteria for occult HCV infection. This patient was male, infected with genotype 1 HCV, had AIDS criteria in the past, carried IL28B genotype CC, and had SVR for 69 months before inclusion in the study. The patient achieved SVR after a 24-week course of treatment with pegylated interferon plus ribavirin (Peg-IFN/RBV). HCV RNA continued to be present in this patient’s PBMCs until the last study visit, with a decreasing number of HCV-positive droplets between the baseline visit and the end of the study (Table 2 and Fig. S1). CD4^+^ counts during follow-up increased from 291 cells/mL to 558 cells/mL at the last visit (Table 2). The antigenomic HCV strand was detected at the first follow-up visit (Table 2).

**Table 2:** Follow-up of the patient detected with HCV RNA.

	Baseline		First visit		Second visit		Third visit	
	Droplets analyzed	Positive droplets	Droplets analyzed	Positive droplets	Droplets analyzed	Positive droplets	Droplets analyzed	Positive droplets
HCV RNA in PBMCs	12.034	12.034	11.380	11.106	14.575	4	13.726	0
HCV RNA in serum	14.615	0	14.197	0	14.353	0	13.295	0
Antigenomic HCV strand	Negative		Positive		Negative		Negative	
CD4 (cells/mL)	290		406		663		558	
Liver stiffness [kPa (IQR)]	3.7 (0.7)		4.6 (1.0)		5.5 (1.0)		5.1 (0.8)	
ALT (U/L)	19		20		29		24	
AST (U/L)	26		24		31		28	
HIV RNA (copies/mL)	<20		<20		<20		<20	

Abbreviations: peripheral blood mononuclear cells (PBMCs); positive droplets (PD); kilopascal (kPA); alanine aminotransferase (ALT); aspartate aminotransferase (AST).

## Discussion

To the best of our knowledge, this is the first study to evaluate the persistence of HCV in PBMCs after achieving long-term SVR using an ultra-sensitive method such as ddPCR. HCV RNA persistence in PBMCs was found in 1 patient who had achieved SVR with sustained clearance of serum HCV RNA for 8 years. Our findings confirm therefore that HCV RNA can persist in PBMCs after long-term SVR, but suggest that this is a rare event. Several studies have described the persistence of HCV RNA in long-term SVR, even after spontaneous resolution of infection^19–25^. In a cohort of 54 patients with long-term SVR, Hedenstierna *et al*. detected HCV RNA in PBMC samples from two patients, and the time between SVR and positivity of samples was 5 and 9 years, respectively. These patients were later HCV RNA-negative in a re-analysis 5 and 4 years later, respectively^20^. Similarly, in a study analyzing whole blood samples from 52 patients with long-term SVR using ultracentrifugation combined with RT-PCR, Lybeck *et al*. found HCV RNA in 2 patients. These determinations were made 8 and 9 years respectively after SVR. A second determination performed months later was HCV RNA-negative^21^. Finally, in a longitudinal study conducted by Garcia-Bengoechea *et al*., 10 SVR patients were followed and showed a gradual decrease in HCV RNA in PBMCs and late disappearance during follow-up, while HCV RNA remained undetectable in serum in all patients^22^. In our study, the patient was HCV RNA-positive in PBMCs 8 years after achieving SVR, but remained undetectable in serum. The patient remained positive at the first visit after baseline, but presented very few positive droplets at the second. Finally, at the last visit, the patient tested HCV RNA-negative. The antigenomic strand was also detected at the first follow-up visit. In line with the studies mentioned, it may be concluded that after achieving SVR, the probability of finding residual HCV RNA decreases over time.

In the context of HIV co-infection, effective treatment with cART suppresses HIV replication and restores the total CD4^+^cell count. Cases have been reported of spontaneous resolution of chronic HCV infection after immune reconstitution based on the use of effective antiretroviral treatment^26–28^. In the patient identified in our study, the progressive clearance of HCV RNA from PBMCs coincided with a significant progressive increase in CD4^+^ cells following SVR, which could suggest that HCV RNA clearance from PBMCs could be associated with the timing of progressive immune reconstitution.

The persistence of HCV RNA in PBMCs could be a potential risk for late relapse, defined as viral rebound after attaining SVR. This has been reported in several studies, which included patients treated with Peg-IFN/RBV or IFN-free therapy^29–33^. In a study including HCV-monoinfected subjects, Desmond *et al*. found late HCV relapse in 1 out of 147 patients who achieved SVR with Peg-IFN/RBV (0.68%). The relapse was identified 46 weeks after achieving SVR^29^. Likewise, in a cohort of 129 transplant patients who achieved SVR, Elmasry *et al*. reported a late HCV relapse rate of 3.8% (n = 5)^30^. In an SVR registry study including 5,433 patients treated with DAAs in combination or not with Peg-IFN/RBV (NCT01457755), Lawitz *et al*. identified 6 patients (0.1%) with late HCV relapse between 217 and 429 days after completion of therapy, confirmed by phylogenetic analysis^31^. Most studies have reported late relapses, even in the first year after achieving SVR^29–33^. In a study analyzing 262 SVR patients treated with Peg-IFN/RBV, Giannini *et al*. described two types of patient with positive HCV RNA after SVR: those who were transiently HCV RNA-positive after a long time with SVR (n = 18), and those who experienced a true late relapse (n = 2) in the first year after achieving SVR^24^. In our study, the median SVR was 51 months, so that we are unable to report possible relapses occurring in the first few months following achievement of SVR. Nonetheless, the patient identified with persistence of HCV RNA did not experience a very late relapse in follow-up, which would be consistent with most studies that have found HCV RNA in long-term SVR patients^20–24^.

Studies that have found residual HCV RNA did not report liver damage or fibrosis progression^20,21,24^. Castillo *et al*. found the antigenomic HCV RNA strand in 15 of 20 SVR patients in a longitudinal study evaluating HCV RNA persistence in paired liver biopsies. Nevertheless, in spite of this evidence of HCV virus replication and persistence, the paired liver biopsies revealed a significant improvement in necroinflammatory activity and liver fibrosis stage after SVR^6^. In our study, the liver stiffness values of this patient did not progress during follow-up, and the patient did not experience transaminase elevations during the study period, which suggests that HCV viral persistence does not correlate with liver injury or liver fibrosis progression.

Nonetheless, given that patients cured of HCV may be organ or blood donors, the possibility of HCV RNA persistence in patients who have achieved SVR may justify further evaluation with highly sensitive procedures. Similarly, the risk of mother-to-child transmission has been associated with the presence of HCV RNA in PBMCs^34,35^. This would be critical in women with SVR who hope to become pregnant, because the persistence of HCV RNA in PBMCs could have important implications for vertical transmission.

Our study presents several limitations that should be noted. First, the HCV RNA detected was not sequenced, so that we were unable to accurately define the phylogeny of the virus. In this respect, although the patient did not engage in risky practices for HCV reinfection, the possibility could not be ruled out. Second, measuring HCV RNA in other compartments, such as the liver, was not available in this study. Finally, this study included only HIV/HCV co-infected patients and the results cannot therefore be extrapolated to series of HCV-monoinfected individuals.

In conclusion, HCV RNA persistence in PBMCs is not a common event in HIV/HCV co-infected patients with long-term SVR after evaluation with an ultra-sensitive procedure such as ddPCR. Only one patient had viral persistence and this did not lead to HCV-related clinical complications, such as late relapse or liver fibrosis progression.

## Supplementary information

Figure S1
