# Supplementary material for: Evaluation of hepatitis C viral RNA persistence in HIV-infected patients with long-term sustained virological response by droplet digital PCR
Source: Sci Rep. 2019 Aug 29;9:12507. doi: 10.1038/s41598-019-48966-9 (PMC6715682; doi:10.1038/s41598-019-48966-9)
Supplement: Supplementary file 1 — Figure S1 [file 41598_2019_48966_MOESM1_ESM.pdf]

Evaluation of hepatitis C viral RNA persistence in HIV-infected patients with long-term sustained virological response by droplet digital PCR

Authors: Mario Frías[1], Antonio Rivero-Juárez[1*], Francisco Téllez[2], Rosario Palacios[3], Álvaro Jiménez-Arranz[4], Juan Antonio Pineda[5], Dolores Merino[6], María Amparo Gómez-Vidal[7], Inés Pérez-Camacho[8], Ángela Camacho[1], Antonio Rivero[1].

Author affiliations:

1. Unidad de Enfermedades Infecciosas. Instituto Maimonides de Investigación Biomédica de Córdoba (IMIBIC). Hospital Universitario Reina Sofía de Córdoba. Universidad de Córdoba. Córdoba, Spain.
2. Unidad Gestión Clínica Enfermedades Infecciosas, Hospital La Línea, AGS Campo de Gibraltar, Cádiz, Spain.
3. Unidad de Enfermedades Infecciosas. Hospital Universitario Virgen de la Victoria. Complejo Hospitalario Provincial de Málaga. Málaga, Spain.
4. Unidad de Genómica. Instituto Maimónides de Investigación Biomédica de Córdoba (IMIBIC). Hospital Universitario Reina Sofía, Universidad de Córdoba. Spain.
5. Unidad de Enfermedades Infecciosas. Hospital Universitario de Valme. Instituto de Biomedicina de Sevilla (iBiS). Sevilla, Spain.
6. Unidad de Enfermedades Infecciosas. Hospitales Juan Ramón Jiménez e Infanta Elena de Huelva. Huelva, Spain.

7. Unidad de Enfermedades Infecciosas. Complejo Hospitalario de Jaén. Jaén, Spain.

8. Unidad de Enfermedades Infecciosas. Hospital de Poniente. El Ejido, Spain.

*Corresponding author: Antonio Rivero-Juárez. Address: Instituto Maimónides de Investigación Biomédica de Córdoba (IMIBIC). Laboratorio de Virología Clínica y Zoonosis, 2ª Planta. Avd. Menendez Pidal s/n. 14004. Cordoba, Spain. Telephone: 0034-957213806. Fax: 0034-957011885. E-mail: arjvet@gmail.com

Figure S1. 1D amplitude plot which shows the droplets. The threshold separating positive and negative droplets was set at 1900.

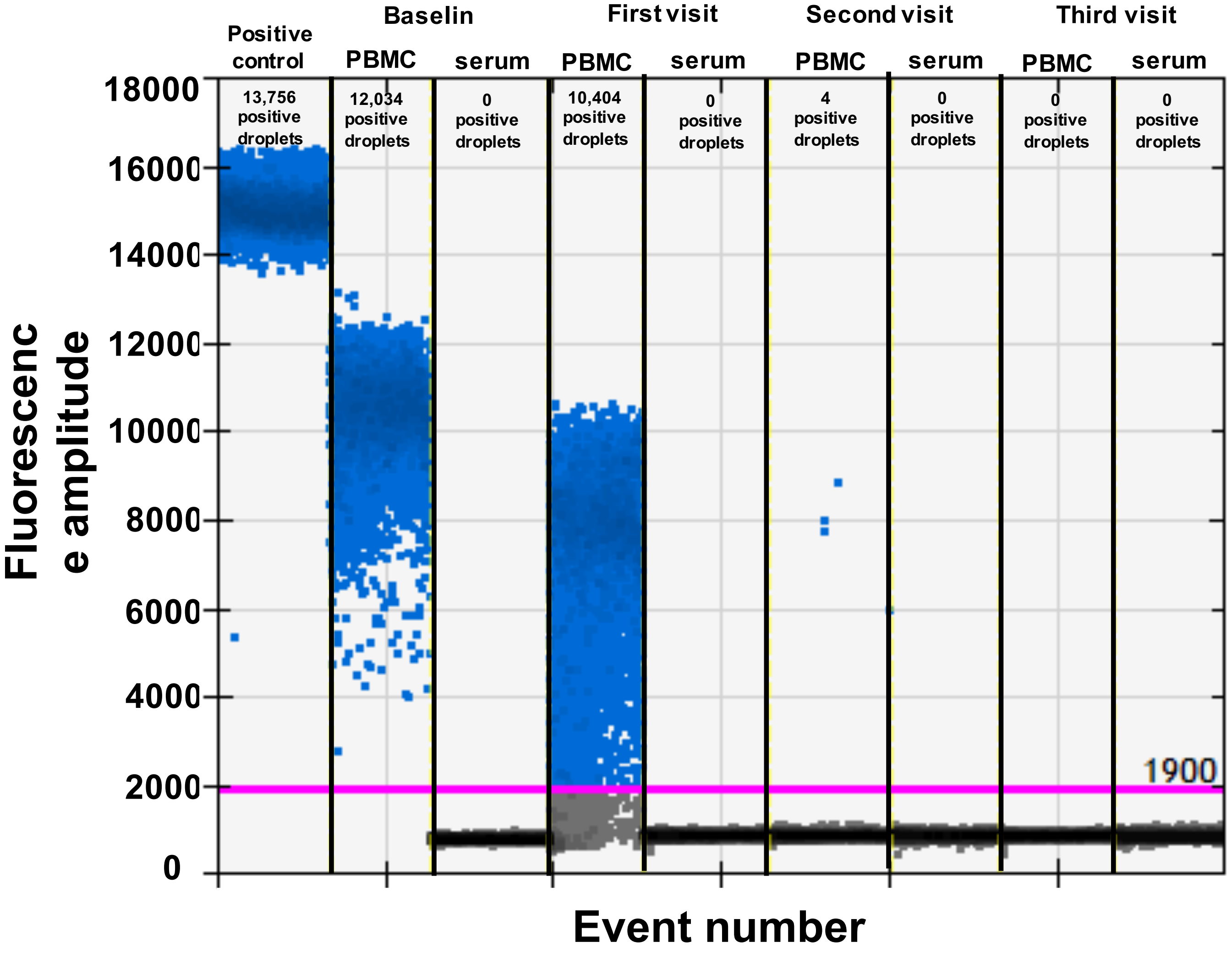

Positive control
Baselin
PBMC
serum
First visit
PBMC
serum
Second visit
PBMC
serum
Third visit
PBMC
serum
13,756 positive droplets
12,034 positive droplets
0 positive droplets
10,404 positive droplets
0 positive droplets
4 positive droplets
0 positive droplets
0 positive droplets
0 positive droplets
18000
16000
14000
12000
10000
8000
6000
4000
2000
0
1900
Fluorescence amplitude
Event number
